# Immuno‐allergic dermatoses in children of 0–5 years old in Kinshasa hospital environment

**DOI:** 10.1002/ski2.332

**Published:** 2024-01-12

**Authors:** Lydie Joelle Nono Seudjip, Christiane Koudoukpo, Adama Traore, Paulo Muntu Bunga

**Affiliations:** ^1^ Departments of Specialties University of Kinshasa Kinshasa Congo; ^2^ Dermatology and Venereology Unit of the University Hospital of Borgou in Parakou University of Parakou Faculty of Medicine Parakou Benin; ^3^ Dermatology Service University of Ouagadougou Health Sciences Training and Research Unit Yalgado Ouedraogo University Hospital Center Ouagadougou Burkina Faso; ^4^ Departments of Pediatrics University of Kinshasa Kinshasa Congo

## Abstract

**Introduction:**

The frequency of immuno‐allergic dermatoses (IAD) is gradually overtaking that of infectious dermatoses in sub‐Saharan Africa. The aim of this study was to identify the epidemioclinical profile and the determinants of IAD in children of 0–5 years old at the University Clinics of Kinshasa (UCK).

**Methods:**

This was a documentary and descriptive study that focused on children from 0 to 5 years old with IAD, over an 11‐year period from 2011 to 2021. Included were children from 0 to 5 years old received in consultation the first time for IAD. The parameters of interest were sociodemographic and clinical. Data was entered and analysed using Excel 2010 software. Ethical and deontological considerations were respected. The value of *p* < 0.05 was the significance threshold.

**Results:**

The frequency of IAD in children aged 0–5 years in the UCK was 17.8%, with a high peak between 2 and 3 years (41.9%) and a female predominance at 54.4%, which represents a sex ratio of 0.8. Prurigo strophulus (42.3%) and atopic dermatitis (22.8%) were the most common IAD. In multivariate analysis, the determinants were significantly the age group of 4–5 years, the rainy season and the child's environment.

**Conclusion:**

Prurigo strophulus and atopic dermatitis were the most frequent IAD in children aged 0–5 years. A holistic care (medical, ecological) of children and their parents may reduce morbidity related to IAD for this age.

## Graphical abstract

Les dermatoses immuno‐allergiques (DIA) constituent une part importante en consultation Dermatopédiatrique (17.8%) à Kinshasa. Les enfants les plus atteints sont âgés de 3 à 5 ans (41.9%). Le prurigo est la DIA plus fréquente. En 11 ans, on a noté une régression de la fréquence des DIA de 17% à 3.3%. La tranche d’âge de 4 à 5 ans, la saison sèche, l'environnement ressortent comme les déterminants des DIA entrae 0–5 ans. L'assainissement du cadre de vie réduirait la recrudescence des dermatoses immuno‐allergiques et/ou les comorbidités dues aux piqûres d'insectes chez les enfants âgés de 0 à 5 ans.

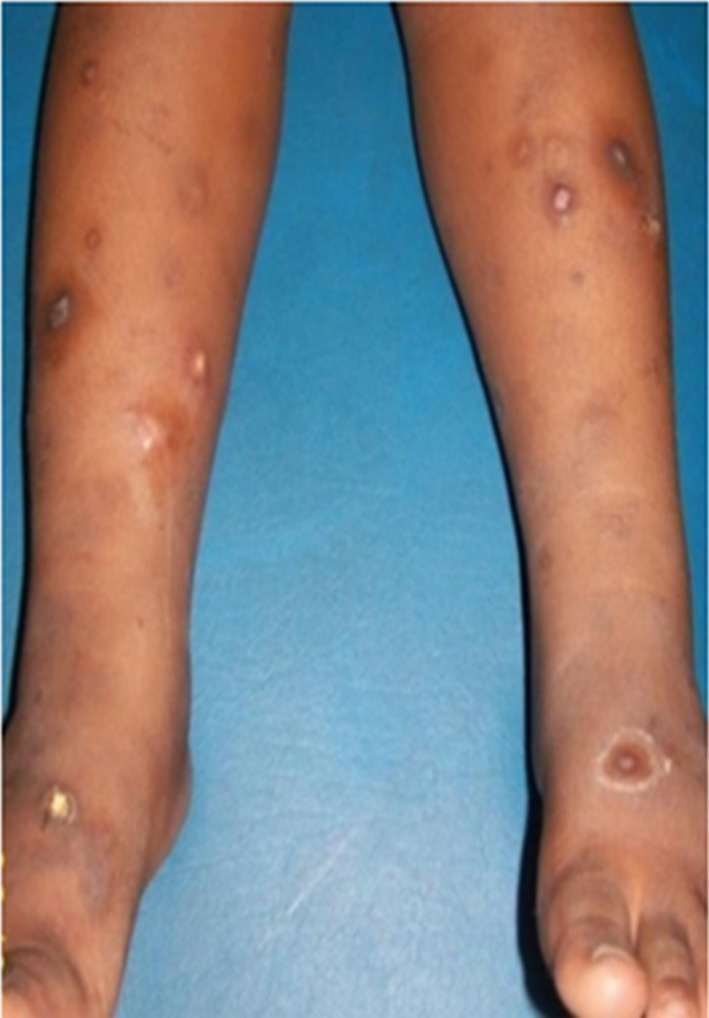

**What's already known about this topic?**

- The authors from West and Central Africa noted an epidemiological reversal of dermatoses in children, as did immuno‐allergic and infectious dermatoses. According to their studies, it turned out that immuno‐allergic dermatoses previously less frequent compared to infectious ones in their environments, become the leader of dermatoses in children. It therefore seemed important to us to note the tendency of the latter in democratic repilicism of the Congo, where a study on the subject has not yet been carried out.

**What does this study add?**

- Our study focused primarily on children aged 0–5 years, which was not the case for the other authors. This age group is very vulnerable in developing countries. While confirming the significant reality of this type of dermatoses in our environment, the main one is prurigo strophulus, contrary to the observation of other authors who have noted a preponderance of atopic dermatitis. Hence the important role of the environment in the genesis of these skin diseases as observed in our study.

## 1 INTRODUCTION

Immuno‐allergic dermatoses (IAD) are nosological group of skin pathologies which include hypersensitivity reaction and spongiform pattern.^1^ Their prevalence in children is the subject of controversy compared to those of infectious dermatoses in developing countries according to certain African authors.^2^, ^3^, ^4^, ^5^ These IAD could be the result of a phenomenon of westernisation of the population's lifestyles and environmental pollution due to progressive industrialisation in African metropolises. A few years ago, infectious dermatoses were topping the list in studies carried out in sub‐Saharan areas^5^, ^6^, ^7^, ^8^, ^9^; this observation is no longer relevant with the emergence of the IAD^1^, ^10^, ^11^ whose chronicity could significantly impact the quality of life of patients. No study has yet been focused exclusively on IAD in the Democratic Republic of Congo. The aim of this study was to identify epidemioclinical characteristics and to identify the determinants of IAD in children of 0–5 years old in the University Clinics of Kinshasa (UCK).

## 2 METHODS

### 2.1 Nature, and period of the study framework

We conducted a documentary descriptive study on children admitted for consultation in the UCK dermatology department, from 2011 to 2021, that is 11 years.

### 2.2 Study population

The files retained were those of the children brought for dermatological consultation to the UCK during the period of study.

### 2.3 Inclusion criteria

The cases included were those of all children from 0 to 5 years old with IAD, received for dermatological consultation during the study period and whose files contained all the parameters of interest.

### 2.4 Parameters of interest

They were socio‐demographic (Age, Sex, Year of consultation, Month of consultation, Residence) and clinical (reason for consultation, lesion site, diagnosis of IAD).

### 2.5 Sampling

We carried out a systematic and documentary review of files containing a diagnosis of IAD in order to collect those of children aged 0–5 years.

### 2.6 Data collection and analysis

The archives enabled us to collect data. We obtained the approval of the ethics committee and the administrative authorities of the hospital in order to carry out this study. From the consultation registers, the records of patients with IAD were extracted, and from this large group those of children aged 0–5 years were selected. The variables of interest were listed on the survey sheets prepared for this purpose. The diagnosis of immuno‐allergic dermatosis was made on the basis of clinical and paraclinical findings. The paraclinical component (biological, histological) was rare in patient files, most diagnoses being strictly clinical.

The data was entered using Excel 2010 software, checked and cleaned. Its statistical analysis consisted of the calculation of means and standard deviations for the quantitative variables. Qualitative variables were expressed in absolute and relative values. Pearson's chi‐square test was used to compare proportions as appropriate. Multivariate logistic regression analysis was performed to identify the determinants of IAD.

Confidentiality and anonymity were respected during data collection and the value of *p* < 0.05 was the significance threshold.

### 2.7 Operational definitions

Immuno‐allergic dermatoses are skin pathologies that combine hypersensitivity with a spongiform pattern dermatosis.^1^ They were represented by the following diagnostic entities: prurigo (prurigo strophulus in the context of this study, which focused on children aged from 0 to 5 years); atopic dermatitis, contact dermatitis, urticaria, eczematid, toxidermia, lichen planus, keratosis pilaris, and diaper rash in infants.

‐ Prurigo (prurigo strophulus): Pruritic papulovesicular dermatitis, inflammatory and recurrent, caused by a hypersensitivity reaction to arthropod bites;
‐ Atopic dermatitis: a chronic inflammatory dermatosis that begins around the age of 2 months and can persist into adulthood. It is characterised by xerosis of the skin associated with eczema lesions;
‐ Contact dermatitis: Cutaneous hypersensitivity reaction to an allergen of variable nature, which progresses through very itchy and well‐limited eruptive flare‐ups on the contact area.
‐ Urticaria: Immuno‐allergic dermatosis characterised by pruritic, migratory and fleeting papules;
‐ Eczématid: an inflammatory erythematosquamous dermatosis similar to atopic dermatitis, of which it is a premonitory sign;
‐ Cutaneous lichen planus: papular, purplish, highly pruritic and often lichenified dermatosis, evolving in flare‐ups;
‐ Toxidermia: skin accidents caused by drugs (ingested, inhaled, injected) taken in non‐toxic doses;
‐ Keratosis pilaris: papular, follicular and dry eruption of childhood evolving on atopic terrain;
‐ Infant W diaper rash: this is a contact dermatitis limited to the diaper area (inner thighs, buttocks, pubis) of infants.

The season was defined as follows:

‐ Dry season: March, June, July and August;
‐ Rainy season: January, February, April, May, September, October, November and December.

The city has 24 communes divided into 4 districts: Lukunga, Mont amba, Funa and Tshangu. The place of residence was the commune where the child lives.

‐ Rural: includes all communes with a rural district (Mont Ngafula, Maluku and Nsele);
‐ Urban‐rural: includes communes that do not have a rural district (all the remaining communes).

## 3 RESULTS

### 3.1 Epidemiological characteristics

#### 3.1.1 Global frequency of dermatosis

Out of 15,559 dermatological consultations at the UCK over the study period, all ages and sexes combined, 1528 (9.82%) concerned patients with IAD. Of this number, 272 were children aged 0–5 years, that is a frequency of 17.8%.

### 3.2 General characteristics of the study population

Over the study period, the general characteristics of IAD in children from 0 to 5 years old have been mentioned in Table 1.

**TABLE 1:** General characteristics of the population (UCK, 2011–2021).

	*n*	%
Age (years)		
[0–1]	88	32.4
[2–3]	**114**	**41.9**
[4–5]	70	25.7
Sex		
Female	**148**	**54.4**
Male	124	45.6
Season		
Dry	116	42.6
Rainy	**156**	**57.4**
Origin (district)		
Mont Amba	**128**	**47.1**
Lukunga	84	30.9
Funa	34	12.5
Tshangu	26	9.6
Year of consultation		
2011	22	8.1
2012	26	9.6
2013	34	12.5
2014	**47**	**17.3**
2015	33	12.1
2016	27	9.9
2017	21	7.7
2018	21	7.7
2019	11	4.0
2020	21	7.7
2021	9	3.3

*Note*: The value in bold represents the highest frequency during the 11 years, period of the study.

The median value of the children's ages was 2 (1–4) years. The extremes were 0–5 years. The majority of these children were between 2 and 3 years old (41.9%).

The distribution according to sex is homogeneous with a slight female predominance (sex ratio = 0.8). Children consulted more during the rainy season (57.4%). The majority of children consulting for a dermatosis lived mainly in the district of Mont Amba (47%) and that of Lukunga (30.9%). According to this data, the year 2014 had the highest number of consultations (17.3%).

### 3.3 Clinical characteristics

#### 3.3.1 Distribution of children according to reasons of consultation

The consultation reasons were represented either by functional signs or by observed dermatological lesions (Table 2).

**TABLE 2:** Reasons of consultation for registered DIA (UCK, 2011–2021).

Reasons of consultation	*n*	%
Functional signs		
Pruritus	227	**83.5**
Pain	4	1.4
Dermatological lesions		
Spots (hyper or hypopigmented)	34	**12.5**
Xerosis	7	2.6
Total	272	100

*Note*: The bold represents the most frequent functional sign and dermatological lesion respectively.

More than three out of four children had pruritus as the reason of consultation (83.5%).

#### 3.3.2 Distribution of children according to location of lesions

The location of lesions was variable, as shown in Table 3.

**TABLE 3:** Location of registered IAD (UCK, 2011–2021).

Lesional sites	*n*	%
Generalised	81	**29.8**
Upper and lower limbs at a time	80	**29.4**
Legs alone	38	14.0
Thorax	21	7.7
Face	19	7.0
Upper limb alone	16	5.9
Head and neck	8	2.9
Seat	5	1.8
External genitalia	4	1.5
Total	272	100

*Note*: The bold represents the most frequent lesional sites, which are generalized and the upper and lower limbs respectively.

The lesions were mostly generalised (29.8%) and localised on the limbs (29.4%).

#### 3.3.3 Diagnostic features

Tables 4 and 5 represent respectively the relationships between the IAD observed/registered in children and the age groups on one hand, the sex and the season on the other hand.

**TABLE 4:** Relationships between registered IAD and age groups (UCK, 2011–2021).

	*n* (%)	Age (%)			*p*
		0–1 year	2–3 years	4–5 years	
Prurigo	120 (44.11)	27 (30.6)	59 (51.7)	34 (48.7)	*0*.*037*
Atopic dermatitis	62 (22.8)	23 (26.1)	26 (22.8)	13 (18.6)	*0*.*022*
Urticaria	45 (16.5)	12 (13.6)	18 (15.7)	15 (21.4)	*0*.*389*
Contact dermatitis	19 (7.0)	7 (8.0)	6 (5.3)	6 (8.6)	*0*.*258*
Eczematid	18 (6.6)	15 (17.0)	1 (0.9)	2 (2.9)	*0*.*001*
Toxidermia	2 (0.7)	0 (0.0)	2 (1.8)	0 (0.0)	‐
Lichen planus	2 (0.7)	2 (2.3)	0 (0.0)	0 (0.0)	‐
Keratosis pilaris	2 (0.7)	0 (0.0)	2 (1.8)	0 (0.0)	‐
W‐shaped diaper rash	2 (0.7)	2 (2.3)	0 (0.0)	0 (0.0)	‐

*Note*: The values in italics represent the number of children with nosological entity (*n*) and the *p*‐value whose significance threshold is less than or equal to 0.05.

**TABLE 5:** Relationships between sex, season and recorded IAD (UCK, 2011–2021).

Dermatoses	Sex		*p*	Season		*p*
	Male *n* = 124 (%)	Female *n* = 148 (%)		Rainy *n* = 156 (%)	Dry *n* = 116 (%)	
Prurigo	56 (45.16)	64 (43.24)	*0*.*548*	72 (45.15)	48 (41.3)	*0*.*478*
Atopic dermatitis	34 (27.4)	28 (18.9)	*0*.*295*	31 (19.9)	31 (26.7)	*0*.*061*
Urticaria	14 (11.2)	32 (21.6)	***0*.*004***	26 (16.66)	19 (16.3)	*0*.*431*
Contact dermatitis	8 (6.5)	11 (7.4)	*0*.*285*	8 (5.1)	11 (9.5)	*0*.*096*
Eczematid	9 (7.3)	9 (6.1)	*0*.*542*	14 (9.0)	4 (3.4)	***0*.*001***
Toxiderma	1 (0.8)	1 (0.7)	*0*.*578*	1 (0.6)	1 (0.9)	*0*.*614*
Lichen planus	1 (0.8)	1 (0.7)	*0*.*578*	1 (0.6)	1 (0.9)	*0*.*614*
Keratosis pilaris	1 (0.8)	1 (0.7)	*0*.*578*	1 (0.6)	1 (0.9)	*0*.*614*
W‐shaped diaper rash	0 (0.0)	2 (1.4)	‐	2 (1.3)	0 (0.9)	‐

*Note*: The values in italics represent the number of children with nosological entity (n) and the *p*‐value whose significance threshold is less than or equal to 0.05. The bold‐italic values represent the statistically significant values of *p*.

It then returns that: Prurigo and atopic dermatitis were the most common dermatoses encountered in children from 0 to 5 years old, respectively in 44.11% and 22.8% of the cases; the frequency of urticaria increased significantly with the age of the children (*p* = 0.037); the frequency of atopic dermatitis decreased significantly with increasing age of the children (*p* = 0.022); Prurigo was significantly more frequent between 2 and 3 years; and eczema more common in children of 0–1 years old.

#### 3.3.4 Distribution of dermatoses according to sex and season

Thus, there was a statistically significant relationship between the occurrence of urticaria and the sex of the child (*p* = 0.004).

Thus, there was a statistically significant relationship between the occurrence of eczema and the season (*p* = 0.001).

After a univariate then multivariate analysis, the determinants of IAD in children aged from 0 to 5 years are recorded in Table 6.

**TABLE 6:** Determinants of immuno‐allergic dermatoses in children of 0–5 years old.

Variables	Univariate analysis		Multivariate analysis	
	*p*	OR (95% CI)	*p*	ORa (95% CI)
Age				
0–1 years		1		1
2–3 years	*0*.*584*	1.56 (0.49–1.96)	*0*.*522*	1.31 (0.47–1.47)
4–5 years	***0*.*016***	2.03 (1.05–3.94)	***0*.*007***	2.04 (1.55–3.97)
Sex				
Male		1		1
Feminine	*0*.*725*	1.09 (0.68–1.76)	*0*.*852*	1.05 (0.64–1.71)
Season				
Rain		1		1
Dried	***0*.*007***	3.55 (1.57–5.52)	***0*.*008***	3.55 (1.95–5.52)
Origin (district)				
Mount Amba		1		1
Lukunga	*0*.*341*	1.56 (0.63–3.89)	*0*.*404*	1.48 (0.59–3.74)
Tshangu	***0*.*017***	2.83 (1.16–4.41)	***0*.*022***	2.73 (1.71–4.21)
Funa	***0*.*033***	2.68 (1.18–4.41)	*0*.*136*	1.65 (0.56–3.83)

*Note*: The values in italics represent the number of children with nosological entity (*n*) and the *p*‐value whose significance threshold is less than or equal to 0.05. The bold‐italic values represent the statistically significant values of *p* in univariate and multivariate analysis.

In multivariate analysis, we found that, the main variables significantly affecting the occurrence of IAD were the age group of 4–5 years (*p* = 0.007), weather conditions, particularly the dried season (*p* = 0.008) and the child's environment (*p* = 0.022).

## 4 DISCUSSION

### 4.1 Epidemiological characteristics of IAD

The aim of this study was to establish the epidemio‐clinical profile, and to identify the determinants of IAD in children aged from 0 to 5 years in a tertiary hospital environment in Kinshasa. Out of a total of 1528 patients seen in dermatology consultations at University Clinics for IAD, 17.8% of the cases were children of 0–5 years old. The frequency of hospital visits and consultations for IAD in children have rarely been reported. In West and Central Africa, IAD represent 32%–37% of cases of dermatoses in general in the paediatric population for an age group beyond 5 years.^2^, ^3^, ^5^ Kouotou et al. found a prevalence of 34.8% in children of 0–9 years old.^1^ The low frequency found in our study could be justified by the age limit of our sample, and the documentary and unicentric nature of the study.

The most affected age group was that of children of 2–3 years old (41.9%), with a predominance in the rainy season (57.4%). Our observation for age is contrary to that of Adegbidi et al.^2^ who noted a high peak in IAD between 0 and 30 months. This could be justified in our work by the early parental and medical care of children while they are still infants. Concerning the resurgence of these IAD in the rainy season, our results corroborate those of Kouotou et al.^1^ in Cameroon during the months of June and July and those of Metelo et al.^12^ who noted a significant increase in mosquitoes during the rainy season. The rainy season in our context is characterised by a both hot and humid weather, which promotes hyper hydration of the skin with disruption of its barrier function, a condition conducive for irritation, hypersensitivity reactions and skin infection.^13^ The predominant sex in our study was female (sex ratio: 0.8). This observation is similar to those of authors (2, 5) from Benin (sex ratio: 0.78) and from Congo (sex ratio F/M: 1.5), unlike Ogunbiyi et al. in Nigeria^14^; who found a sex ratio close to 1. Could the female sex also be a predisposing factor for IAD?.^15^ Most of the IAD were observed among the population from the Mont Amba District. This due to the presence of UCK in this district. Contrary to the Beninese and Cameroonian results,^1^, ^2^ we noted a maximum peak of IAD in 2014 (17.3%), followed by a decrease in this frequency during the following 7 years. This would be the consequence of better multidisciplinary and specialised care offered by the hospital.

### 4.2 Clinical features of IAD

Pruritus was the main reason for consultation (83.5%) and lesions were generalised in 29.8% of cases.

**FIGURE 1:**
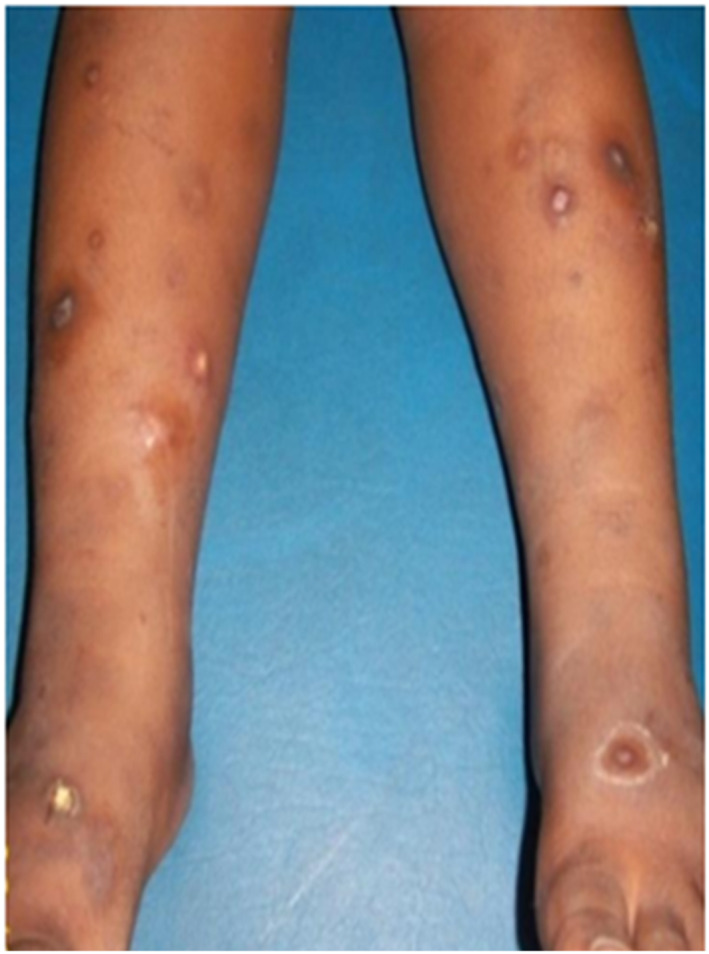
Prurigo: excoriated, hyperpigmented lesions with epidermal collarette on the legs.

**FIGURE 2:**
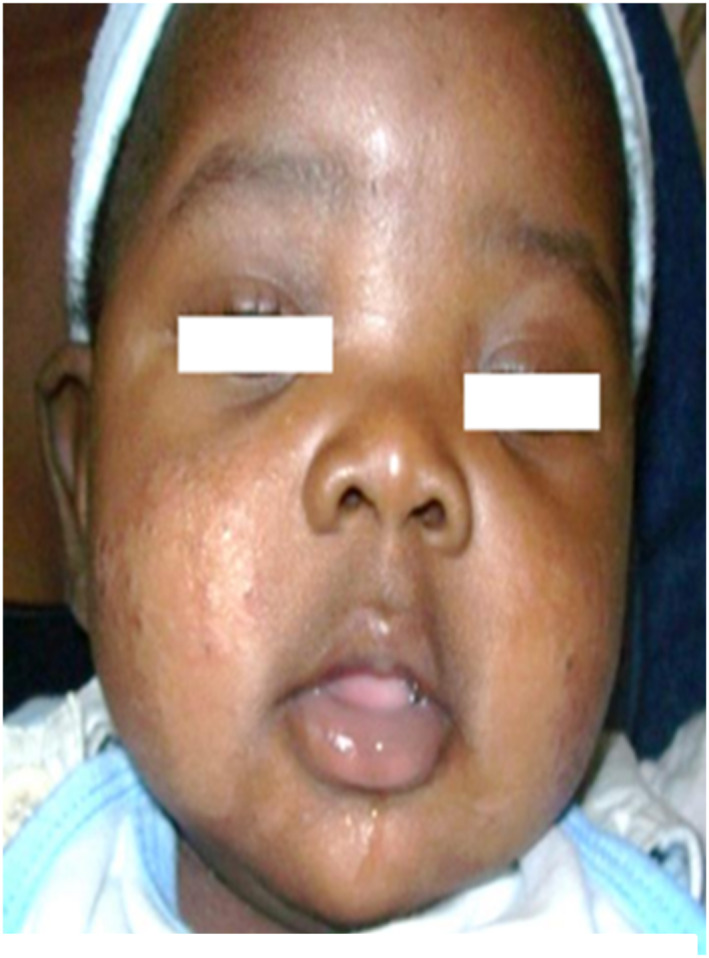
Atopic dermatitis: xerotic and cracked plaques, slightly scaly on the cheeks.

Prurigo (Figure 1) was the first IAD observed (44.11%), followed by atopic dermatitis (Figure 2) (22.8%). The frequency of prurigo increased with age, unlike that of atopic dermatitis, and this very significantly (*p* = 0.037; *p* = 0.022), with high peaks respectively between 2 and 3 years and 0–1 year of age.

Our observations on prurigo were similar to those of Ahogo et al.^16^ in Ivory Coast and Kouotou et al.^1^ in Cameroon. The extent of prurigo in our study could be linked to the promiscuity and precarious state of the environment, favourable for the reproduction of biting insects, in this case mosquitoes, but also to children's access to open air progressively as they grow.

Concerning atopic dermatitis, our finding is contrary to those of French,^17^ Beninese^2^ and Cameroonian^1^ studies where dermatitis was the first or third IAD. Knowing that atopic dermatitis is a dermatitis of early expression,^18^ which is the case in the present study, its low frequency could denote under‐informed parents about this condition and prior self‐medication before a medical consultation. Likewise, the westernisation of the lifestyle of the populations also leads to the occurrence of immuno‐allergic affections. Our frequency for eczematids was not comparable to that of Al‐Mendalawi et al.^19^ who found 0.3%–0.7% of cases. This dermatosis is considered to be a precursor to atopic dermatitis. Its very significant importance (*p* = 0.001) in the age group of 0–1 year could explain more in this study the link that exists between the two dermatoses immuno‐allergiques. As in the literature and with its natural history, we have noted a decreasing and statistically significant evolution of atopic dermatitis according to age.^18^

**FIGURE 3:**
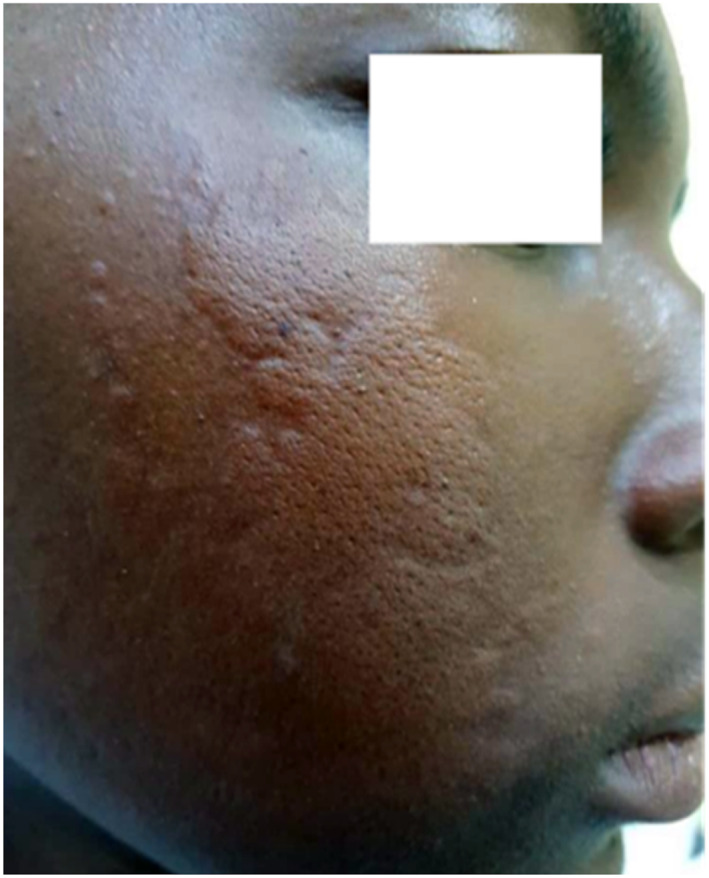
Urticaria: oedematous plaque, orange peel appearance on the cheek.

**FIGURE 4:**
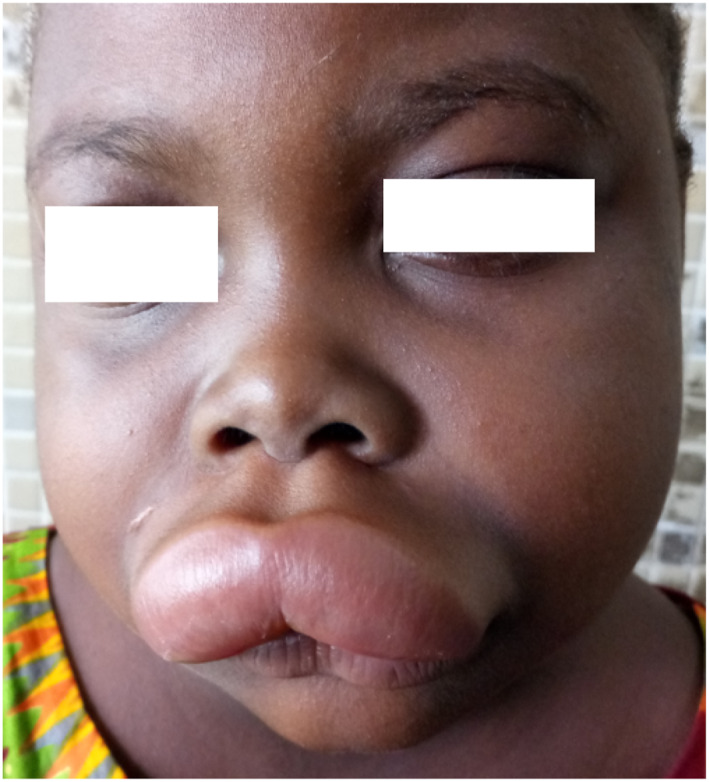
Urticaria: Quincke's oedema of the upper lip.

Urticaria (Figures 3 and 4) was statistically linked to sex (*p* = 0.004), with a female predominance (21.6%) and a frequency of 11.2%. Higher value than many Turkish^20^ and African^1^, ^3^, ^5^ authors. The low frequency found in our study, may in part be due to the age limit of our sample and the unicentric nature of the study.

Eczematid was very significantly linked to the season (*p* = 0.001), with an increase in the rainy period (9%). This association had not been found in a previous study. Nevertheless, the hot and humid weather is a factor favouring the occurrence of atopic dermatitis lesions,^21^ and consequently those of eczematid, indicator of atopic eczema.^22^

Finally, it emerged from the present study that in multivariate analysis, the main determinants of IAD in children aged from 0 to 5 years were the age group of 4–5 years, the dry season and the district of Tshangu, which respectively represented 2, 4 and 3 times the risk of causing IAD.

### 4.3 Limits of the study

‐ The retrospective nature and monocentric context of this study are epidemioclinical data loss biases that do not allow extrapolation of results to the general population of children aged 0–5 years
‐ The diagnosis of immunoallergic dermatoses was clinical in most cases.

Nevertheless, our study has some positive points.

‐ This original study of IAD is the first of its kind in the Democratic Republic of Congo.
‐ The results will form a database for future perspectives.

## 5 CONCLUSION AND RECOMMENDATIONS

Immuno‐allergic dermatoses in children from 0 to 5 years old in Kinshasa's tertiary hospital environment account for a significant proportion (17.8%) in paediatric dermatology consultations, although their frequencies are decreasing over the years. Prurigo was more frequent. Environmental sanitation could reduce the resurgence of these dermatoses and comorbidities linked to insect bites. By way of recommendations, in order to halt the increase in the frequency of IAD, we recommend a large‐scale multicentre study aimed at identifying most of the determinants of IAD, which will enable targeted preventive and curative action to be taken, including the promotion of good therapeutic education in the context of these dermatoses.

## CONFLICT OF INTEREST STATEMENT

None to declare.

## AUTHOR CONTRIBUTIONS

**Lydie Joelle Nono Seudjip**: Conceptualization (equal); formal analysis (equal); funding acquisition (equal); investigation (equal); methodology (equal); project administration (equal); writing—original draft (equal); writing—review and editing (equal). **Christiane Koudoukpo**: Resources (equal); supervision (equal); visualization (equal). **Adama Traore**: Supervision (equal); validation (equal); visualization (equal). **Paulo Muntu Bunga**: Resources (equal); supervision (equal); validation (equal); visualization (equal); writing—original draft (equal).

## ETHICS STATEMENT

Not applicable.

## Data Availability

The data underlying this article will be shared on reasonable request to the corresponding author.
